# Investigating the Association between Wood and Charcoal Domestic Cooking, Respiratory Symptoms and Acute Respiratory Infections among Children Aged Under 5 Years in Uganda: A Cross-Sectional Analysis of the 2016 Demographic and Health Survey

**DOI:** 10.3390/ijerph17113974

**Published:** 2020-06-04

**Authors:** Katherine E. Woolley, Tusubira Bagambe, Ajit Singh, William R. Avis, Telesphore Kabera, Abel Weldetinsae, Shelton T. Mariga, Bruce Kirenga, Francis D. Pope, G. Neil Thomas, Suzanne E. Bartington

**Affiliations:** 1 Institute of Applied Health Research, University of Birmingham, Edgbaston, Birmingham B15 2TT, UK; KEW863@student.bham.ac.uk (K.E.W.); TVB850@alumni.bham.ac.uk (T.B.); G.N.Thomas@bham.ac.uk (G.N.T.); 2 School of Geography, Earth and Environmental Sciences, University of Birmingham, Edgbaston, Birmingham B15 2TT, UK; A.Singh.2@bham.ac.uk (A.S.); F.Pope@bham.ac.uk (F.D.P.); 3 International Development Department, University of Birmingham, Edgbaston, Birmingham B15 2TT, UK; W.R.Avis@bham.ac.uk; 4 College of Science and Technology, University of Rwanda, Avenue de l’Armee P.O. Box 3900, Rwanda; kaberacris@yahoo.fr; 5 Ethiopian Public Health Institute, Addis Ababa P.O. Box 1242, Ethiopia; abelweldetinsae@gmail.com; 6 Makerere University Lung Institute, College of Health Sciences, Mulago Hospital, Kampala P.O. Box 7749, Uganda; sheltontmariga@gmail.com (S.T.M.); brucekirenga@yahoo.co.uk (B.K.)

**Keywords:** acute respiratory infection, biomass fuel, household air pollution, respiratory symptoms, Uganda

## Abstract

*Background*: Household air pollution associated with biomass (wood, dung, charcoal, and crop residue) burning for cooking is estimated to contribute to approximately 4 million deaths each year worldwide, with the greatest burden seen in low and middle-income countries. We investigated the relationship between solid fuel type and respiratory symptoms in Uganda, where 96% of households use biomass as the primary domestic fuel. *Materials and Methods*: Cross-sectional study of 15,405 pre-school aged children living in charcoal or wood-burning households in Uganda, using data from the 2016 Demographic and Health Survey. Multivariable logistic regression analysis was used to identify the associations between occurrence of a cough, shortness of breath, fever, acute respiratory infection (ARI) and severe ARI with cooking fuel type (wood, charcoal); with additional sub-analyses by contextual status (urban, rural). *Results*: After adjustment for household and individual level confounding factors, wood fuel use was associated with increased risk of shortness of breath (AOR: 1.33 [1.10–1.60]), fever (AOR: 1.26 [1.08–1.48]), cough (AOR: 1.15 [1.00–1.33]), ARI (AOR: 1.36 [1.11–1.66] and severe ARI (AOR: 1.41 [1.09–1.85]), compared to charcoal fuel. In urban areas, Shortness of breath (AOR: 1.84 [1.20–2.83]), ARI (AOR: 1.77 [1.10–2.79]) and in rural areas ARI (AOR: 1.23 [1.03–1.47]) and risk of fever (AOR: 1.23 [1.03–1.47]) were associated with wood fuel usage. *Conclusions*: Risk of respiratory symptoms was higher among children living in wood compared to charcoal fuel-burning households, with policy implications for mitigation of associated harmful health impacts.

## 1. Introduction

Globally, almost 3 billion people utilize biomass fuels such as wood, charcoal, animal dung, and plant residues for everyday domestic activities including cooking and heating [1,2]. These fuels are typically burned in simple stoves or open fires, with inefficient combustion associated with the release of hazardous gases and particles such as carbon monoxide (CO), nitrogen dioxide (NO_2_), and particulate matter (PM) [1,3]. The arising pollutants are the major contributors to Household Air Pollution (HAP), responsible for almost 4 million deaths worldwide each year, with the greatest burden in low and middle-income country (LMIC) settings, including sub-Saharan Africa [2]. Chronic HAP exposure associated with biomass fuel combustion is recognized to be detrimental to human health, with consistent evidence for an association with respiratory and cardiovascular disease conditions including acute respiratory tract infections (ARIs) [4], asthma [5], chronic obstructive pulmonary disease (COPD) [6], lung cancer [7], tuberculosis [6], stroke, ischemic heart disease [2] and metabolic and cognitive health [8]. Moreover, families living in rural households and those engaged in subsistence agriculture are recognized to experience a higher HAP morbidity and mortality burden, compared to those living in urban LMIC settings [9,10,11].

Uganda is a landlocked country in East Africa, with an increasing population of 43 million and a gross domestic product (GDP) per capita of approximately $643 USD in 2018, 24.2% of which is generated by the agricultural sector [12]. Uganda has a population density of 231 people per km^2^ with an urban population of 10 million (23.3%) in 2018, with growth of 6.2% since 2017; a significant proportion of which live in or around the Ugandan capital Kampala. The life expectancy of Uganda is 62.5 years, with an under five-mortality rate of 46.4 children per 1,000 live births [13]; both comparable to other East African countries.

Prevailing reliance upon biomass energy in sub-Saharan Africa also presents broader implications for achieving sustainable development, including fiscal costs imposed by morbidity and mortality, opportunity costs of fuel collection and environmental degradation through deforestation and carbon dioxide emissions [14]. In Uganda, East Africa, it is estimated that 92% of the total energy demand comes from biomass, with 96% of domestic cooking being undertaken on firewood and charcoal, resulting in annual wood consumption of 20 million tons [15]. HAP is a major priority risk factor for Uganda, responsible for 2.93% of deaths, including 1.47% of deaths due to respiratory disease [16]. Access to cleaner fuels, such as electricity and Liquid Petroleum Gas (LPG), is limited due to logistical and financial supply barriers particularly high capital infrastructure costs, most notably in rural areas; therefore contributing to prevailing health and social inequity [15].

Limited existing epidemiological evidence from Uganda suggests an association between use of biomass fuels for cooking and incidence of chronic respiratory and cardiovascular disease [17,18]; however few studies have investigated the relationship between solid fuel usage for cooking and incidence of respiratory symptoms in pre-school aged children. Most existing research has focused upon health impacts among those living in rural areas and to the best of our knowledge there is no previous study of respiratory symptoms associated with charcoal and wood fuel cooking among a nationally representative population sample, including both rural and urban biomass fuel households. The present study therefore aims to identify the association between risk of respiratory symptoms (cough, fever and short rapid breaths), ARI and severe ARI (Infection of the upper or lower respiratory tract presenting with cough, shortness of breath and fever) [19] with charcoal and wood fuel cooking among children aged under 5 years in Uganda, using data obtained from the population-based Demographic and Health Survey (DHS).

## 2. Materials and Methods

### 2.1. Setting and Study Design

Data for this cross-sectional study were obtained from the most recently available nationally representative Ugandan DHS [20], a population level survey implemented by the Uganda Bureau of Statistics (UBOS, supported by USAID, UNICEF, UNFPA) from 20 June to 28 December 2016 [21]. Two-stage stratified sampling was applied to identify eligible residential households across 697 enumeration areas (average 130 households) from 112 Districts and 15 regions in Uganda. From 20,791 eligible households, 18,506 resident ever-married women age 15–49 years were interviewed (98% response rate) [21]. Institutional living arrangements (e.g., boarding schools, police camps, army barracks, and hospitals) were excluded; as were households with no response at the time of fieldwork completion.

Survey questionnaires were modified from those within the Phase VII DHS Program model, adapted to reflect the population and health issues relevant to Uganda. Information for this study was obtained from the (i) household questionnaire; comprising information on household structure, socio-demographic and housing characteristics, including domestic cooking fuel type, and; (ii) children’s questionnaire; including questions on maternal and child health outcomes and lifestyle characteristics. All survey fieldwork was undertaken within the participant’s home, by trained local fieldworkers supervised by senior staff, with data entry directly to tablet computers transferred to the UBOS central processing office by a secure internet system.

### 2.2. Ethical Approval and Authorization

Ethical approval for primary data collection was provided by the Uganda Ministry of Health. The investigators obtained the anonymised, aggregate data from the publicly available DHS online data archive [20] with authorization granted for data access for this current investigation on 16 June 2019.

### 2.3. Data Variables

#### 2.3.1. Measures of Respiratory Symptoms and Acute Respiratory Infections

To assess respiratory symptoms, maternal respondents were asked if each of their children aged under 5 years had experienced the following symptoms within the two weeks prior to the survey: (i) a cough (ii) short rapid breaths or difficulty breathing (iii) a fever; each categorized and modelled as binary outcome measures (yes, no). A composite measure was created for both ARI and severe ARI, reflecting the presence of respiratory symptoms with or without fever, with each separately modelled as a binary health outcome measure (yes, no). ARI was classified as present of cough and short rapid breaths/difficulty [22], whereas severe ARI was composed of the presence of all three of these symptoms, (e.g., cough, short rapid breaths/difficulty breathing and fever) [23].

#### 2.3.2. Measure of Exposure to HAP

Among those households in which cooking activities were performed, self-reported cooking fuel types were identified from the household dataset and categorized as “cleaner fuels” (electricity, LPG, natural gas, biogas); “Solid biomass fuels and kerosene” (kerosene, coal/lignite, charcoal, wood, straw/shrubs/grass, agricultural crop, animal dung) or other fuel types. Those mother-child pairs living in households reporting wood and charcoal fuel cooking were extracted for further analyses.

#### 2.3.3. Child and Maternal Characteristics

Characteristics of household children comprised; age (0–11, 12–23, 24–35, 36–48, 48–59 months), sex (male, female), birthweight (kg, by maternal recall), weight for height (z score), mode of delivery (caesarean, vaginal), birth order (first, not first born), breastfeeding status (ever, never), vitamin A supplementation in the last 6 months (yes, no), iron supplementation (yes, no). Those children with diagnosed mild, moderate or severe anaemia (*n* = 2139) were excluded from further analyses, due to anaemia being a known factor for increased ARI risk [24], which could not be accounted for in the adjusted analyses due to the high quantity of missing data (2139/15405; 13.9%). Maternal characteristics included age (15–24, 25–35, 36–49 years) and highest attained educational level (none, primary, secondary/higher).

#### 2.3.4. Household and Geographical Characteristics

Household characteristics were accounted for by the following variables: number of household members, indoor household smoking (yes, no), cooking location (inside, outdoors). Season at the time of DHS contact was determined from the month of interview and classified as dry (June to August) or wet (September to November) using information from the Central Intelligence Agency (CIA) fact book [25]. The five category DHS wealth index was used as a measure of household level socio-economic status (lowest, low, middle, high, highest). This composite measure reflects household ownership of selected assets (e.g., television, bicycle, car), dwelling characteristics (e.g., source of drinking water, sanitation facilities, types of cooking fuel, and floor material), with assessment of relative wealth category calculated by principle components analysis.

Contextual characteristics comprised: place of residence (rural, urban), and country region (Kampala, South Buganda, North Buganda, Busoga, Bukedi, Bugisu, Teso, Karamoja, Lango, Acholi, West Nile, Bunyoro, Tooro, Ankole, Kigez). DHS classifies rural and urban area, as per the country of survey; in this case Uganda uses enumeration areas are defined as being rural or urban. Urban areas are defined as officially approved cities, municipalities, town councils and town boards [21], at the time which the survey was undertaken.

### 2.4. Data Analysis

All data processing, manipulation and analyses was performed using R studio [26]. Descriptive statistics were summarized by number of cases (*n*), percentages (%) (categorical variables) and median and interquartile range (IQR) (continuous variables). The association between fuel type (wood vs. charcoal) and respiratory health outcomes (cough, fever, short rapid breaths or ARI/severe ARI), was determined through multivariable logistic regression analysis; reporting the odds ratio (OR), 95% Confidence interval (95% CI) and level of significance (*p*-value). Univariable forward selection was used to determine variables for inclusion in the adjusted analysis. Covariates include, child’s age, sex, birth order, mode of delivery, vitamin A supplementation, breastfeeding, iron supplementation, maternal age, maternal education, wealth index, household smoking, cooking location, number of household remembers, season, place of residence, region. Statistical significance in the adjusted model was set at *p* < 0.05. Model collinearity was checked using variance inflation factors (VIF function in R). The primary analysis was performed upon the whole dataset, with subsequent sub-analyses by rural and urban area status respectively.

## 3. Results

A total of 19,588 households were included in the 2016 DHS survey in Uganda, at a 98% response rate. Data were collected for 15,522 children under five years. A total 0.3% of children resided in a households using ‘cleaner’ fuels (0.2%—electricity, 0.1%—LPG, 0.04%—Biogas) and 99.3% using biomass as the main source of cooking fuel; the majority (78.6%) using wood, charcoal (21.4%). Of these children, we excluded those with diagnosed anaemia (13.9%). Among charcoal and wood fuel households, 93% had complete information regarding the outcome variables: presence of cough and shortness of breath within the last two weeks (Table 1). Of this study population, the median child age was 30 months (IQR: 14–45) with 81.7% residing in a rural location.

Within rural study areas, wood fuel use was used by a higher proportion of respondents (87.5%) compared to urban areas (35.9%), when compared to charcoal fuel. The rural study population had a median age of 30 months (IQR: 14–45 months) compared to 31 months (IQR: 15–45 months) in the urban population.

### 3.1. Shortness of Breath in the Past Two Weeks

Shortness of breath was reported among 17.7% of children living in wood fuel households compared to 13.0% in charcoal households (*p* < 0.001) (Table 2). After adjustment for confounding factors (wealth index, season, region, place of residence household size, maternal level of education, breastfeeding status, mode of delivery, vitamin A supplementation and iron supplementation) shortness of breath was significantly associated with cooking fuel type, with an observed increased risk associated with wood fuel (adjusted OR (AOR): 1.33; 95% CI: 1.10–1.60; *p* < 0.01) compared to charcoal cooking. Other factors associated with significantly increased risk in adjusted analyses were male sex (AOR: 1.13; 95% CI: 1.02–1.26; *p* < 0.05) and vaginal delivery (AOR: 1.39; 95% CI: 1.08–1.83; *p* < 0.05).

### 3.2. Cough in the Past Two Weeks

Cough was reported among 40.0% of children residing in charcoal cooking households, compared to 39.1% in wood fuel households (*p* = 0.412). In the adjusted analysis, risk of cough was significantly higher among those living in wood (AOR: 1.15; 95% CI: 1.00–1.33; *p* < 0.05), compared to charcoal cooking fuel households. Other observed factors associated with increased risk of cough were vaginal delivery (AOR: 1.27; 95%CI: 1.06–1.54; *p* < 0.05), vitamin A supplementation (AOR: 1.67; 95% CI: 1.53–1.83; *p* < 0.001), outdoor cooking (AOR: 1.13; 95% CI: 1.02–1.25; *p* < 0.05) and dry season (OR: 1.18; 95% CI: 1.08–1.28; *p* < 0.001).

### 3.3. Fever in the Past Two Weeks

Of those children who resided in wood fuel households, 35.2% were reported to exhibit a fever compared to 23.4% in charcoal cooking households (*p* < 0.001), which remained significant after adjustment for confounding factors (AOR: 1.26; 95% CI: 1.08–1.48; *p* < 0.01). In adjusted analyses, risk of fever was also significantly associated with vitamin A supplementation (AOR: 1.48; 95% CI: 1.34–1.63; *p* < 0.001) and vaginal delivery (AOR: 1.38; 95% CI: 1.11–1.73); *p* < 0.01), with reduced risk among children of mothers with a higher level of education (AOR: 0.80; 95% CI: 0.71–0.91: *p* < 0.001) or highest wealth index (AOR: 0.64; 95%CI: 0.51–0.81; *p* < 0.001). Risk of fever was highest among those children aged 12–24 months, with a reduced risk among infants in the first year of life (AOR 0.67; 95% CI: 0.59–0.77: *p* < 0.001) and those between 24 months and 5 years of age.

### 3.4. ARI (Shortness of Breath and Cough in the Past Two Weeks) and Severe ARI (Shortness of Breath, Cough and Fever in the Past Two Weeks)

ARI was observed within 15.1% of children in wood fuel compared to 11.1% of charcoal fuel households. In the adjusted analysis wood fuel was observed to be associated with increased ARI risk (AOR: 1.36; 95% CI: 1.11–1.66); *p* < 0.01), as was vaginal delivery (AOR: 1.37 95% CI: 1.04–1.83; *p* < 0.05) and vitamin A supplementation (AOR: 1.58; 95% CI: 1.40–1.78; *p* < 0.001).

Among wood fuel households, 9.5% of children were reported to have shown symptoms of severe ARI compared to 5.6% of those living in charcoal fuel households (*p* < 0.001). After adjustment for confounding factors, wood fuel was observed to be associated with increased severe ARI risk (AOR: 1.41; 95% CI: 1.09–1.85; *p* < 0.05), as was male sex (AOR: 1.19; 95% CI: 1.04–1.38; *p* < 0.05), vaginal delivery (AOR: 1.51 95% CI: 1.04–2.28; *p* < 0.05) and vitamin A supplementation (AOR: 1.67; 95% CI: 1.43–1.96; *p* < 0.001). Older children aged between 36 months and 5 years also had reduced ARI and severe ARI risk compared to those aged between 12 and 23 months.

### 3.5. Urban and Rural Population

Regression analyses were repeated separately for rural and urban areas. Among those living in urban areas, shortness of breath was reported among 18.9% of children in wood fuel households compared to 1.09% in charcoal fuel households (*p* < 0.001), with wood fuel being observed to increase the risk of shortness of breath (AOR: 1.84; 95% CI: 1.20–2.83; *p* < 0.01) and ARI (AOR: 1.77; 95% CI: 1.10–2.79; *p* < 0.05) compared to charcoal fuel (Table 3). Conversely, no significant association was observed between cooking fuel type and risk of cough, fever or severe ARI, although absolute effect sizes were consistent with those for the combined analysis. In addition, within the rural population there was only an observed association between wood fuel type and risk of fever (AOR: 1.23; 95% CI: 1.03–1.47; *p* < 0.05) and ARI (AOR: 1.27; 95% CI: 1.01–1.59; *p* < 0.05) (Table 3).

## 4. Discussion

Globally, almost half of the world’s population cook with solid biomass fuels; associated with pollutant concentrations which typically exceed WHO Indoor Air Quality Guidelines [27]. Although traditional combustion of charcoal cooking fuel is recognized to emit lower air pollutant concentrations compared to firewood, there is a paucity of evidence regarding respiratory health benefits of charcoal biomass fuel alternatives and no previous evidence from Uganda, where over 96% of the population use biomass fuels as the primary domestic energy source [15]; compounded by health and social inequality as a result of barriers to cleaner fuel access.

This observational, nationally-representative population based study, with a large sample size (*n* = 15,522) and high response rate (98%) has identified that wood fuel cooking is associated with increased individual risk of respiratory symptoms and ARI risk among children aged under 5 years living in biomass fuel households in urban and rural settings in Uganda. Children are recognized to be at increased vulnerability to HAP exposure and ARI is a leading causes of death among those aged under 5 years worldwide, contributing to an estimated 1.9–2.2 million deaths worldwide each year [28]; therefore prevention could have wide reaching health and fiscal benefits.

Our observations regarding the increased respiratory risks associated with wood compared to charcoal biomass fuel are broadly consistent with current evidence from low income contexts [29,30]. Sana et al. [31] showed that women living within an urban population in Burkina Faso had increased risk of cough among those using wood, compared to charcoal cooking. Taylor and Nakai [32] also observed ARI prevalence to be higher for children in homes with wood stoves compared to charcoal stoves in Sierra Leone, and overall ARI prevalence was higher than reported previously. However, this study was undertaken in the rainy season in Sierra Leone, which is recognized to influence the occurrence of ARI and ARI symptoms [32]. Our study showed a significant association with season (two seasons observed over 6 months), with the dry season (June to August) increasing the risk of cough in both urban and rural settings.

Our study has a number of limitations; the DHS survey includes questions only about fuel type and we did not have available information regarding other fuels used to light the stove or mixed fuel use. Charcoal is recognized to be difficult to ignite, therefore a starting fuel is typically required, which may comprise wood, straw or crop residues in rural areas, with plastics or kerosene more likely to be used in urban areas, therefore influencing exposure to specific air pollutants include volatile organic compounds (VOCs) [31]. However, wood combustion is consistently associated with high concentrations of particulate matter [33,34,35,36,37], which is likely to be the dominant pollutant in biomass fuel domestic settings and which has a causal relationship with ARI risk in other sub-Saharan settings [38,39,40]. Furthermore, detailed information was not available for wood and charcoal fuel types (such as tree species, seasoning), family cooking practices or fuel usage patterns and mixing, which limits comparison of findings between study populations, or those which rely upon fuel type as a proxy for air pollutant exposure.

ARI risk is also recognised to be caused by cumulative long-term pollutant exposure [32], which cannot be determined in this cross-sectional study. However, evidence suggests that fuel choices and cooking remain relatively consistent among biomass fuel households in low-income contexts [4]. Therefore, similar levels of chronic exposure would be expected for those using a specific biomass fuel type. Another contributory factor is household smoking, which has a low prevalence in Uganda (16.4%) [41], and within this study 13.3% of children resided in a household with a smoking household member; reflecting the important contribution of HAP to respiratory disease risk in this context. Although outdoor cooking and ventilation has been shown to decrease HAP levels [42], it may not be below the WHO level; which may explain why there is an increased risk of cough with outdoor cooking. Our use of DHS data at the household and individual maternal and child levels enabled adjustment for a wide range of socio-demographic, household and contextual factors known to be associated with the risk of respiratory symptoms, including socio-economic status (wealth index), season, region, household size, maternal level of education, breastfeeding status, mode of delivery and vitamin A supplementation and birth order. However, these factors may be subject to recall bias and detailed information was not available for co-inhabitation with livestock, cooking activity patterns, or other sources of domestic or ambient pollution; however, our findings reflect the widespread use of fuel type as a proxy indicator and which is meaningful and easily communicated to families in this context.

Biomass is the primary domestic fuel in sub-Saharan Africa (76.7%) [43], which remains the world region with the highest burden arising from household air pollution. In the context of limited availability of cleaner fuels mitigation measures have potential large scale public health benefit for primary prevention of respiratory symptoms and disease in this setting. The energy ladder presents the transition phase from solid biomass and readily collected fuels (such as wood, dung, straw) through processed natural fuels (e.g., charcoal) to supplied “cleaner” fuels (e.g., electricity, LPG); associated with shift to an industrialized energy economy [44]. In experimental setting, charcoal, although still presenting some health risks [31] was associated with lower levels of CO, CO_2_ and PM_2.5_ compared to wood [45]; however these vary according to moisture content, wood type and circumstances of combustion (among other factors). In addition, similar to charcoal are briquettes, another potential transition towards cleaner fuel, are associated with lowered HAP levels, but still exceed the WHO guidelines levels [46]; and require further research into the respiratory benefit.

DHS data is based upon self-reported respiratory symptoms, which can be subject to recall bias, and our primary outcome measure does not represent an objective clinical ARI diagnosis. Therefore, future research is need to determine the air quality and health benefits arising from differences in fuel type, utilising objective air quality assessment and including information on chronic health conditions, cooking activity behaviours, household layout stove design and ventilation. In addition, future DHS surveys and research which includes detailed individual level information on pre-existing health conditions would enable assessment of the role of pre-existing vulnerability, such as the recognised associations between TB [28], HIV [47], ARI severity [47] and poverty [48,49]. Furthermore, epidemiological studies evaluating the impacts of solid biomass fuels should capture the wider environmental impacts, physical hazards and opportunity costs associated with wood collection and charcoal production [50]; along with the occurrence of childhood morbidities affecting further life chances.

Our novel findings suggest potential benefits arising from transition from wood to charcoal fuels in low-income contexts where financial and logistical barriers limit access to cleaner domestic fuels; particularly among those families living in urban areas. A harm mitigation policy approach could be adopted to incentivize transition away from wood to cleaner biomass fuels (such as processed high quality charcoal and briquettes) whilst supporting traditional cooking activity practices and therefore enabling sustained uptake. Major national initiatives to facilitate cleaner fuel use have been frequently unsuccessful due to a lack of socio-cultural considerations, health awareness, education, affordability (fuels, stoves) and supply chain issues [51,52]. In order for improvement in respiratory health to occur, policy makers need to focus on clean fuel switching taking into consideration other confounding factors and vulnerabilities (wealth, breastfeeding, mothers education) found within this study and others [31,53,54,55], which can be modified through policy.

## 5. Conclusions

Replacing wood with charcoal cooking fuel may reduce the incidence of ARI respiratory symptoms shortness of breath, fever, cough, ARI and severe ARI in children aged under 5 years in urban Uganda. Although charcoal is typically more expensive than wood, public health policies applying a harm mitigation approach in contexts which lack cleaner fuel access, may reduce the HAP associated morbidity and mortality burden among young children in this setting.

## Figures and Tables

**Table 1:** Biomass fuel status (all study households) by respiratory outcome measures (frequency, percentage).

	Shortness of Breath (N = 12,161)			Fever (N = 12,179)			Cough (N = 12,163)			ARI (N = 12,145)			Severe ARI (N = 11,967)		
	No (N = 10,137) *n* (%)	Yes (N = 2024) *n* (%)	*p* Value	No (N = 8207) *n* (%)	Yes (N = 3972) *n* (%)	*p* Value	No (N = 7385) *n* (%)	Yes (N = 4778) *n* (%)	*p* Value	No (N = 10414) *n* (%)	Yes (N = 1731) *n* (%)	*p* Value	No (N = 10937) *n* (%)	Yes (N = 1030) *n* (%)	*p* Value
**Household cooking fuel**			<0.001			<0.001			0.412			<0.001			<0.001
Charcoal	2315 (22.8)	345 (17.0)		2044 (24.9)	624 (15.7)		1595 (21.6)	1062 (22.2)		2360 (22.7)	294 (17.0)		2482 (22.7)	147 (14.3)	
Wood	7822 (77.2)	1679 (83.0)		6163 (75.1)	3348 (84.3)		5790 (78.4)	3716 (77.8)		8054 (77.3)	1437 (83.0)		8455 (77.3)	883 (85.7)	
**Child age (months)**			<0.001			0.022			<0.001			<0.001			<0.001
0–11	2183 (26.1)	516 (29.5)		1893 (28.1)	809 (23.9)		1669 (27.7)	1031 (25.1)		2272 (26.4)	427 (28.4)		2417 (26.6)	241 (27.1)	
12–23	1799 (21.5)	435 (24.9)		1377 (20.4)	860 (25.4)		1212 (20.1)	1023 (24.9)		1854 (21.5)	378 (25.2)		1955 (21.6)	234 (26.3)	
24–35	1951 (23.3)	435 (24.9)		1540 (22.8)	847 (25.0)		1379 (22.9)	1006 (24.5)		2001 (23.2)	382 (25.4)		2128 (23.5)	223 (25.1)	
36–47	2068 (24.7)	323 (18.5)		1629 (24.2)	766 (22.6)		1470 (24.4)	922 (22.5)		2107 (24.5)	280 (18.6)		2184 (24.1)	171 (19.2)	
48–59	369 (4.4)	40 (2.3)		305 (4.5)	103 (3.0)		285 (4.7)	124 (3.0)		373 (4.3)	35 (2.3)		386 (4.3)	20 (2.2)	
**Child sex**			0.031			0.142			0.445			0.100			0.010
Male	5003 (49.4)	1052 (52.0)		4047 (49.3)	2015 (50.7)		3654 (49.5)	2398 (50.2)		5150 (49.5)	893 (51.6)		5402 (49.4)	552 (53.6)	
**Birth order**			0.773			<0.001			0.054			0.687			0.024
Not first born	8013 (79.0)	1613 (79.7)		6416 (78.2)	3220 (81.1)		5843 (79.1)	3783 (79.2)		8240 (79.1)	1377 (79.5)		8628 (78.9)	837 (81.3)	
**Mode of delivery ¥**			0.002			<0.001			0.177			0.006			<0.001
Caesarean delivery	596 (5.9)	84 (4.2)		525 (6.4)	156 (3.9)		168 (2.3)	79 (1.7)		607 (5.9)	73 (4.2)		641 (5.9)	34 (3.3)	
**Birth weight (kg) ¥**			0.580			0.024			0.208			0.312			0.642
Median (IQR)	3.3 (3.0, 3.8)	3.3 (2.9, 3.9)		3.2 (3.0, 3.8)	3.3 (3.0, 4.0)		3.2 (3.0, 3.8)	3.3 (3.0, 3.9)		3.3 (3.0, 3.8)	3.2 (2.9, 3.9)		3.3 (3.0, 3.8)	3.2 (2.9, 3.9)	
**Weight/Height Z score ¥**			0.704			0.075			0.478			0.785			0.625
Median (IQR)	19 (−54, 86)	24 (−62, 92)		22 (−51, 90)	18 (−63, 83)		20 (−52, 90)	22 (−58, 85)		20 (−55, 86)	24 (−63, 92)		21 (−53, 87)	23 (−70, 92)	
**Vitamin A supplementation in the last 6 months ¥**			<0.001			<0.001			<0.001			<0.001			<0.001
No	4544 (45.1)	747 (37.1)		3780 (46.4)	1514 (38.3)		3546 (48.4)	1744 (36.7)		4672 (45.1)	613 (35.6)		4855 (44.7)	345 (33.7)	
**Iron supplementation¥**			0.173			0.002			0.806			0.179			0.003
No	9425 (93.7)	1873 (92.9)		7652 (94.1)	3660 (92.6)		6854 (93.6)	4447 (93.5)		9687 (93.7)	1600 (92.8)		10186 (93.8)	938 (91.4)	
**Breastfeeding status**			<0.001			0.037			<0.001			0.131			<0.001
Ever breastfed	9923 (97.9)	1990 (98.3)		8031 (97.9)	3901 (98.2)		4760 (64.5)	2949 (61.7)		10194 (97.9)	1704 (98.4)		10710 (97.9)	1015 (98.5)	
**Maternal age (years)**			0.019			0.086			0.029			0.020			0.719
15–24	3377 (33.3)	692 (34.2)		2721 (33.2)	1358 (34.2)		2444 (33.1)	1625 (34.0)		3459 (33.2)	601 (34.7)		3660 (33.5)	348 (33.8)	
25–35	4976 (49.1)	1028 (50.8)		4109 (50.1)	1906 (48.0)		3620 (49.0)	2387 (50.0)		5125 (49.2)	873 (50.4)		5384 (49.2)	514 (49.9)	
36–49	1784 (17.6)	304 (15.0)		1377 (16.8)	708 (17.8)		1321 (17.9)	766 (16.0)		1830 (17.6)	257 (14.8)		1893 (17.3)	168 (16.3)	
**Maternal level of education**			<0.001			<0.001			0.029			<0.001			<0.001
No education	1271 (12.5)	322 (15.9)		1033 (12.6)	560 (14.1)		988 (13.4)	605 (12.7)		1312 (12.6)	281 (16.2)		1390 (12.7)	179 (17.4)	
Primary	6315 (62.3)	1245 (61.5)		4932 (60.1)	2642 (66.5)		4634 (62.7)	2927 (61.3)		6488 (62.3)	1061 (61.3)		6773 (61.9)	663 (64.4)	
Secondary/Higher	2551 (25.2)	457 (22.6)		2242 (27.3)	770 (19.4)		1763 (23.9)	1246 (26.1)		2614 (25.1)	389 (22.5)		2774 (25.4)	188 (18.3)	
**Household wealth index**			<0.001			<0.001			0.021			<0.001			<0.001
Lowest	2512 (24.8)	638 (31.5)		1833 (22.3)	1318 (33.2)		1920 (26.0)	1232 (25.8)		2595 (24.9)	554 (32.0)		2716 (24.8)	382 (37.1)	
Middle	1977 (19.5)	380 (18.8)		1618 (19.7)	744 (18.7)		1432 (19.4)	928 (19.4)		2037 (19.6)	318 (18.4)		2146 (19.6)	168 (16.3)	
Highest	1697 (16.7)	224 (11.1)		1561 (19.0)	361 (9.1)		1140 (15.4)	778 (16.3)		1721 (16.5)	193 (11.1)		1813 (16.6)	86 (8.3)	
**Household smoking status**			0.427			0.839			0.706			0.672			0.293
Yes	1326 (13.1)	278 (13.7)		1078 (13.1)	527 (13.3)		982 (13.3)	624 (13.1)		1369 (13.1)	234 (13.5)		1434 (13.1)	147 (14.3)	
**Household cooking location ¥**			0.631			<0.001			0.041			0.082			0.333
Indoors	7648 (75.5)	1505 (74.6)		6103 (74.4)	3051 (77.0)		982 (13.3)	624 (13.1)		7865 (75.6)	1274 (73.7)		8241 (75.4)	754 (73.3)	
**Number of household members**			0.513			<0.001			0.062			0.422			0.018
1–4	2988 (29.5)	585 (28.9)		2511 (30.6)	1078 (27.1)		2129 (28.8)	1446 (30.3)		3060 (29.4)	505 (29.2)		3252 (29.7)	263 (25.5)	
5–8	5358 (52.9)	1097 (54.2)		4350 (53.0)	2110 (53.1)		3921 (53.1)	2538 (53.1)		5511 (52.9)	940 (54.3)		5780 (52.8)	575 (55.8)	
9–27	1791 (17.7)	342 (16.9)		1346 (16.4)	784 (19.7)		1335 (18.1)	794 (16.6)		1843 (17.7)	286 (16.5)		1905 (17.4)	192 (18.6)	
**Survey season**			<0.001			<0.001			<0.001			0.097			<0.001
Wet	4075 (40.2)	770 (38.0)		3284 (40.0)	1565 (39.4)		3068 (41.5)	1776 (37.2)		1945 (18.7)	271 (15.7)		4373 (40.0)	396 (38.4)	
**Place of residence**			<0.001			<0.001			0.068			0.003			<0.001
Urban	1913 (18.9)	306 (15.1)		1748 (21.3)	472 (11.9)		1310 (17.7)	910 (19.0)		1945 (18.7)	271 (15.7)		2055 (18.8)	142 (13.8)	

N = total number of observations, *n* = Number of observation for categorical variables % = column percentages for categorical variables, Median = Continuous variable median, IQR = Interquartile Range, *p* value = Chi-Squared for categorical variables, and Kruskal Wallis for Continuous variables. ¥ contains missing observations; Received Vitamin A = 84 (0.7%); Birth weight = 3750 (30.8%); Height/Weight = 9900 (81.3%); Cooking location = 17 (0.1%); Mode of delivery = 49 (0.4%); Taking iron supplements = 90 (0.7%-Fever).

**Table 2:** Adjusted Logistic regression analysis for the whole study population (N = 13,266).

	Shortness of Breath (N = 12,161)			Cough (N = 12,163)			Fever (N = 12,179)			ARI (N= 12,145)			Severe ARI (N = 11,967)		
Predictor (N)	% †	OR	CI	% †	OR	CI	% †	OR	CI	% †	OR	CI	% †	OR	CI
**Household cooking fuel**															
Charcoal (2660)	13.0	*Ref*		40.0	*Ref*		23.4	*Ref*		11.1		*Ref*	5.6	*Ref*	
Wood (9501)	17.7	**1.33 ^b^**	**1.10–1.60**	39.1	**1.15 ^a^**	**1.00–1.33**	35.2	**1.26 ^b^**	**1.08–1.48**	15.1	**1.36 ^b^**	**1.11–1.66**	9.5	**1.41 ^a^**	**1.09–1.85**
**Child age (months) (3972)**															
0–11 (2707)	19.1	1.09	0.94–1.27	38.2	0.83 ^b^	0.73–0.93	29.9	**0.67 ^c^**	**0.59–0.77**	15.8	1.04	0.89–1.22	9.1	0.93	0.76–1.13
12–23 (2237)	19.5	*Ref*		45.8	*Ref*		38.4	*Ref*		16.9		*Ref*	10.7	*Ref*	
24–35 (2387)	8.2	0.94	0.80–1.09	42.4	0.89	0.79–1.01	35.5	**0.85 ^a^**	**0.75–0.97**	16.0	0.95	0.81–1.12	9.5	0.87	0.71–1.06
36–47 (2395)	13.5	**0.64 ^c^**	**0.54–0.76**	38.5	**0.77 ^c^**	**0.68–0.87**	32.0	**0.72 ^c^**	**0.63–0.83**	11.7	**0.66 ^c^**	**0.55–0.78**	7.3	**0.65 ^c^**	**0.52–0.80**
48–59 (408)	9.8	**0.47 ^c^**	**0.32–0.66**	30.3	**0.55 ^c^**	**0.43–0.69**	25.2	**0.50 ^c^**	**0.38–0.65**	8.6	**0.51 ^c^**	**0.35–0.73**	4.9	**0.47 ^b^**	**0.28–0.74**
**Child sex**															
Female (6106)	15.9	*Ref*		38.9			32.0	*Ref*		14.8		*Ref*	7.9	*Ref*	
Male (6055)	17.4	**1.13 ^a^**	**1.02–1.26**	39.6	1.06	0.98–1.15	33.2	1.07	0.98–1.18	13.7	1.12	1.00–1.25	9.3	**1.19 ^a^**	**1.04–1.38**
**Mode of delivery ¥**															
Caesarean section (680)	12.4	*Ref*		36.9			22.9	*Ref*		14.5		*Ref*	5.0	*Ref*	
Vaginal delivery (11,298)	16.9	**1.39 ^a^**	**1.08–1.83**	39.5	**1.27 ^a^**	**1.06–1.54**	33.2	**1.38 ^b^**	**1.11–1.73**	10.7	**1.37 ^a^**	**1.04–1.83**	8.8	**1.51 ^a^**	**1.04–2.28**
**Vitamin A supplementation in last 6 months ¥**															
No (5291)	14.1	*Ref*		33.0			28.6	*Ref*		11.6		*Ref*	6.6	*Ref*	
Yes (6792)	8.7	**1.47 ^c^**	**1.31–1.64**	44.3	**1.67 ^c^**	**1.53–1.83**	35.8	**1.48 ^c^**	**1.34–1.63**	16.3	**1.58 ^c^**	**1.40–1.78**	10.1	**1.67 ^c^**	**1.43–1.96**
**Breastfeeding status**															
Ever (11,932)	16.7	*Ref*		39.4	*Ref*		32.7	*Ref*		14.3		*Ref*	8.7	*Ref*	
Never (247)	13.7	0.81	0.50–1.26	32.0	**0.71 ^a^**	**0.51–0.98**	28.7	0.91	0.63–1.29	10.9	0.76	0.45–1.22	6.2	0.71	0.33–1.34
**Maternal age (years) (3972)**															
15–24 (4079)	17.0	*Ref*		39.9	*Ref*		33.3	*Ref*		14.8		*Ref*	8.7	*Ref*	
25–35 (6015)	17.1	1.07	0.93–1.23	39.7	0.98	0.88–1.09	31.7	0.99	0.88–1.11	14.6	1.01	0.87–1.17	8.7	1.00	0.83–1.21
36–49 (708)	14.6	0.97	0.80–1.17	36.7	0.92	0.79–1.06	34.0	1.02	0.87–1.19	12.3	0.91	0.74–1.11	8.2	0.95	0.74–1.22
**Maternal level of education**															
No education (1593)	20.2	0.95	0.79–1.14	38.0	0.92	0.79–1.06	35.2	0.98	0.84–1.15	17.6		*Ref*	11.4	0.86	0.67–1.10
Primary (7560)	16.5	*Ref*		38.7	*Ref*		34.9	*Ref*		14.1	0.95	0.78–1.16	8.9	*Ref*	
Secondary/Higher (3008)	16.2	1.06	0.92–1.23	41.4	1.02	0.91–1.14	27.5	**0.80 ^c^**	**0.71–0.91**	13.0	1.05	0.90–1.23	6.9	0.87	0.71–1.06
**Household wealth index**															
Lowest (3151)	20.3	*Ref*		39.1	*Ref*		41.8	*Ref*		17.6		*Ref*	12.3	*Ref*	
Low (2678)	16.7	1.05	0.89–1.24	36.9	0.99	0.87–1.13	35.3	0.96	0.84–1.10	14.2	1.03	0.87–1.23	9.0	1.04	0.84–1.28
Middle (2362)	16.1	1.04	0.87–1.25	39.3	1.05	0.91–1.21	31.5	1.07	0.92–1.24	13.5	0.98	0.81–1.20	7.3	0.96	0.75–1.23
High (2066)	16.3	1.09	0.89–1.33	41.4	1.06	0.91–1.25	29.3	0.97	0.81–1.14	13.9	1.08	0.87–1.34	7.7	1.14	0.87–1.48
Highest (1922)	11.7	0.78	0.59–1.04	40.6	0.92	0.74–1.13	18.8	**0.64 ^c^**	**0.51–0.81**	10.1	0.75	0.55–1.00	4.5	0.68	0.46–1.02
**Household cooking location** **¥**															
Indoors (9154)	16.4	*Ref*		38.7	*Ref*		33.3	*Ref*		13.9		*Ref*	8.4	*Ref*	
Outdoors (3008)	17.2	1.02	0.89–1.17	41.1	**1.13 ^a^**	**1.02–1.25**	30.3	1.08	0.96–1.21	15.2	1.06	0.91–1.22	9.3	1.13	0.94–1.35
**Number of household members (3972)**															
1–4 (3589)	16.4	*Ref*		40.4	*Ref*		30.0	*Ref*		14.2		*Ref*	7.5	*Ref*	
5–8 (6460)	17.0	0.97	0.85–1.11	39.3	1.00	0.90–1.10	32.7	0.94	0.84–1.06	14.6	0.98	0.85–1.13	9.0	1.07	0.89–1.28
8–27 (2130)	16.0	0.89	0.74–1.06	37.3	**0.87 ^a^**	**0.75–0.99**	36.8	0.90	0.78–1.05	13.4	0.89	0.74–1.07	9.2	1.01	0.80–1.28
**Survey season**															
Wet (4649)	15.9	*Ref*		36.7	*Ref*		32.3	*Ref*		13.6		*Ref*	8.3	*Ref*	
Dry (7330)	17.1	1.09	0.98–1.23	41.0	**1.18 ^c^**	**1.08–1.28**	32.8	1.03	0.93–1.13	14.7	1.09	0.97–1.23	8.8	1.11	0.95–1.29

^a^*p* < 0.05; ^b^ *p* < 0.01; ^c^ *p* < 0.001, † Number of cases, with percentage of cases to non-cases with each category, OR = Adjusted odd ratio, CI = 95% confidence interval. Ref. = reference category. ¥ Variable has missing observations: Vitamin A supplementation in last 6 months = 84 (0.7%), Mode of delivery = 49 (0.4%). Not associated in the multivariate model (*p* > 0.05) = Birth order, Place of residence, Household smoking, Taking Iron pills, sprinkles or syrup.

**Table 3:** Adjusted logistic regression analyses for urban and rural study sub-populations (N = 2426, N = 10,840).

	Shortness of Breath						Cough						Fever						Severe ARI					
	Urban (N = 2219)			Rural (N = 12,161)			Urban (N = 2220)			Rural (N = 12,163)			Urban (N = 2220)			Rural (N = 9959)			Urban (N = 2197)			Rural (N = 9770)		
***Predictors (N)***	*% †*	*OR*	*CI*	*% †*	*OR*	*CI*	*% †*	*OR*	*CI*	*% †*	*OR*	*CI*	*% †*	*OR*	*CI*	*% †*	*OR*	*CI*	*% †*	*OR*	*CI*	*% †*	*OR*	*CI*
**Household Cooking fuel**																								
Charcoal	10.9	*Ref.*		13.0	*Ref.*		40.2	*Ref*		40.0	*Ref.*		18.4	*Ref.*		29.0	*Ref.*		4.6	*Ref.*		6.8	*Ref.*	
Wood	18.9	**1.84 ^b^**	**1.20–2.83**	17.7	1.22	0.99–1.50	42.4	1.16	0.84–1.58	39.1	1.22	0.99–1.50	26.3	1.35	0.93–1.95	36.0	**1.23 ^a^**	**1.03–1.47**	9.8	1.77	0.97–3.20	9.4	1.33	0.99–1.80
**Child age (months)**																								
0–11	14.0	0.91	0.61–1.36	19.1	1.13	0.96–1.33	32.9	**0.61 ^c^**	**0.46–0.82**	38.2	0.89	0.78–1.01	18.2	0.61 ^b^	0.43–0.87	32.3	0.69 ^c^	0.60–0.80	5.9	0.73	0.41–1.28	9.7	0.97	0.78–1.20
12–23	15.6	*Ref.*		19.5	*Ref.*		47.6	*Ref.*		45.8	*Ref.*		27.8	*Ref.*		41.1	*Ref*		8.0	*Ref.*		11.4	*Ref*	
24–35	13.7	0.87	0.59–1.30	18.2	0.94	0.80–1.11	42.4	0.90	0.68–1.20	42.2	0.90	0.79–1.03	21.6	**0.69 ^a^**	**0.49–0.96**	38.5	0.89	0.77–1.03	6.7	0.88	0.51–1.51	10.1	0.87	0.70–1.08
36–47	13.2	0.89	0.59–1.32	13.5	**0.60 ^c^**	**0.50–0.72**	42.8	0.95	0.72–1.27	38.5	**0.74 ^c^**	**0.64–0.85**	21.9	**0.71 ^a^**	**0.50–1.00**	34.2	**0.73 ^c^**	**0.63–0.85**	5.6	0.73	0.41–1.28	7.6	**0.64 ^c^**	**0.51–0.81**
48–59	7.5	0.48	0.18–1.09	9.8	**0.47 ^c^**	**0.31–0.68**	33.8	0.70	0.41–1.18	30.3	**0.52 ^c^**	**0.40–0.68**	12.5	**0.40 ^a^**	**0.18–0.82**	28.4	**0.52 ^c^**	**0.39–0.69**	5.0	0.73	0.21–2.03	4.9	**0.43 ^b^**	**0.25–0.72**
**Child sex**																								
Female	12.7	*Ref.*		15.9	*Ref.*		39.8	*Ref.*		38.9	*Ref.*		20.5	*Ref.*		34.7	*Ref.*		5.4	*Ref.*		8.5	*Ref.*	
Male	14.9	1.31	0.99–1.73	17.4	1.11	0.98–1.24	42.2	1.15	0.94–1.40	39.6	1.04	0.95–1.14	22.0	1.15	0.90–1.46	35.6	1.06	0.96–1.17	7.6	**1.51 ^a^**	**1.02–2.26**	9.6	1.14	0.98–1.33
**Mode of delivery ¥**																								
Caesarean	9.2	*Ref.*		16.9	*Ref.*		40.2	*Ref.*		36.9	*Ref.*		8.7	*Ref.*		26.1	*Ref.*		4.3	*Ref.*		5.3	*Ref.*	
Vaginal	90.8	1.20	0.76–1.97	12.4	**1.46 ^a^**	**1.07–2.03**	41.1	1.13	0.82–1.56	39.5	**1.33 ^a^**	**1.06–1.68**	91.3	1.28	0.85–1.99	35.6	**1.42 ^b^**	**1.10–1.87**	6.7	1.19	0.61–2.56	9.3	**1.64 ^a^**	**1.04–2.74**
**Vitamin A supplementation in last 6 months ¥**																								
No	12.2	*Ref.*		14.1	*Ref.*		34.4	*Ref.*		33.0	*Ref.*		16.3	*Ref.*		31.2	*Ref.*		5.6	*Ref.*		6.9	*Ref.*	
Yes	15.2	1.32	0.98–1.78	18.7	**1.49 ^c^**	**1.32–1.69**	46.4	**1.72 ^c^**	**1.40–2.13**	44.3	**1.68 ^c^**	**1.52–1.85**	25.2	**1.69 ^c^**	**1.30–2.20**	38.3	**1.46 ^c^**	**1.31–1.62**	7.1	1.30	0.86–2.00	10.8	**1.74 ^c^**	**1.47–2.06**
**Breastfeeding status**																								
Ever	13.9	*Ref.*		16.7	*Ref.*		41.2	*Ref.*		39.4	*Ref.*		21.4	*Ref.*		35.2	*Ref.*		6.5	*Ref.*		9.1	*Ref.*	
Never	9.3	0.71	0.21–1.86	13.7	0.84	0.49–1.36	32.1	0.56	0.27–1.10	32.0	0.76	0.53–1.10	15.1	0.86	0.33–1.96	32.5	0.93	0.62–1.37	3.8	0.91	0.14–3.22	6.8	0.67	0.28–1.36
**Maternal age (years)**																								
15–24	15.6	*Ref.*		17.0	*Ref.*		43.4	*Ref.*		39.9	*Ref.*		24.3	*Ref.*		35.2	*Ref.*		8.1	*Ref.*		11.1	*Ref.*	
25–35	13.7	0.79	0.56–1.12	17.1	1.14	0.97–1.32	40.5	**0.70 ^b^**	**0.54–0.90**	39.7	1.04	0.93–1.18	19.3	0.75	0.55–1.03	34.7	1.02	0.90–1.16	5.8	**0.56 ^a^**	**0.34–0.92**	9.1	1.11	0.91–1.36
26–49	10.3	**0.48 ^a^**	**0.27–0.83**	14.6	1.08	0.88–1.33	37.6	**0.66 ^a^**	**0.45–0.95**	36.7	0.98	0.83–1.15	21.9	0.87	0.55–1.36	36.2	1.04	0.87–1.23	5.5	0.51	0.23–1.06	7.5	1.05	0.80–1.37
**Maternal level of education**																								
None	10.8	0.90	0.44–1.76	15.9	0.95	0.78–1.15	42.6	1.20	0.71–2.01	38.0	0.90	0.77–1.05	8.9	0.65	0.33–1.21	14.8	1.01	0.86–1.19	15.5	1.27	0.50–2.94	17.7	0.84	0.64–1.08
Primary	48.0	*Ref.*		61.5	*Ref.*		41.5	*Ref.*		38.7	*Ref*		48.1	*Ref.*		69.0	*Ref.*		47.9	*Ref.*		67.0	*Ref.*	
≥Secondary	41.2	0.76	0.55–1.06	22.6	1.14	0.97–1.34	40.3	0.93	0.74–1.18	41.4	1.05	0.92–1.19	43.0	0.69 ^a^	0.52–0.92	16.2	**0.82 ^b^**	**0.71–0.94**	36.6	0.75	0.47–1.21	15.3	0.90	0.71–1.12
**Household wealth index**																								
Lowest	24.4	*Ref.*		20.3	*Ref.*		41.6	*Ref.*		39.1	*Ref.*		35.7	*Ref.*		42.3	*Ref.*		15.4	*Ref.*		12.1	*Ref.*	
Low	11.7	0.72	0.33–1.51	16.7	1.06	0.90–1.26	31.8	0.94	0.54–1.64	36.9	0.99	0.87–1.13	23.3	0.97	0.52–1.79	35.9	0.96	0.84–1.11	7.0	1.03	0.39–2.53	9.1	1.04	0.84–1.29
Middle	19.6	1.27	0.68–2.34	16.1	1.01	0.83–1.23	44.1	1.11	0.68–1.82	39.3	1.04	0.90–1.21	28.2	1.15	0.66–1.98	31.8	1.05	0.89–1.23	7.8	1.06	0.46–2.38	7.2	0.94	0.72–1.22
High	12.4	0.76	0.41–1.39	6.3	1.13	0.91–1.40	38.6	1.01	0.63–1.61	41.4	1.09	0.92–1.30	22.2	0.81	0.48–1.37	30.8	0.99	0.82–1.18	6.8	0.92	0.41–2.04	7.9	1.15	0.86–1.53
Highest	11.6	1.08	0.56–2.10	11.7	**0.64 ^a^**	**0.45–0.89**	42.0	1.20	0.72–1.99	40.6	**0.78 ^a^**	**0.61–1.00**	17.1	0.80	0.45–1.42	22.3	**0.58 ^c^**	**0.43–0.76**	4.5	0.89	0.37–2.17	4.7	**0.58 ^a^**	**0.34–0.95**
**Household cooking location¥**																								
Indoors	14.4	*Ref.*		16.4	*Ref.*		42.1	*Ref.*		38.7	*Ref.*		21.1	*Ref.*		35.3	*Ref.*		6.6	*Ref.*		8.7	*Ref.*	
Outdoors	13.0	0.87	0.64–1.18	17.2	1.06	0.91–1.24	39.4	0.95	0.76–1.18	41.1	**1.20 ^b^**	**1.06–1.35**	21.5	0.97	0.74–1.27	34.2	1.11	0.97–1.26	6.3	0.95	0.61–1.47	10.6	1.15	0.94–1.41
**Number of household members**																								
1–4	14.1	*Ref.*		16.4	*Ref.*		41.8	*Ref.*		39.9	*Ref.*		22.2	*Ref.*		32.6	*Ref.*		5.9	*Ref*		8.0	*Ref*	
5–8	14.2	1.02	0.74–1.39	17.0	0.96	0.83–1.12	41.2	0.98	0.78–1.23	39.7	0.99	0.89–1.12	20.4	0.79	0.60–1.05	35.2	0.97	0.86–1.10	6.9	1.13	0.72–1.79	9.5	1.05	0.86–1.29
9–27	11.1	0.65	0.36–1.12	16.0	0.92	0.76–1.11	37.3	0.97	0.67–1.40	36.7	**0.85 ^a^**	**0.73–0.98**	22.1	0.82	0.52–1.27	38.7	0.92	0.78–1.08	6.2	0.75	0.33–1.58	9.5	1.03	0.80–1.32
**Survey season**																								
Wet	12.1	*Ref.*		15.9	*Ref.*		35.2	*Ref.*		36.7	*Ref.*		16.8	*Ref.*		36.0	*Ref.*		4.7	*Ref.*		9.2	*Ref.*	
Dry	15.0	1.23	0.90–1.67	17.1	1.07	0.95–1.22	45.2	**1.62 ^c^**	**1.31–2.01**	41.0	**1.11 ^a^**	**1.01–1.22**	24.5	**1.51^b^**	**1.15–1.99**	34.6	0.96	0.86–1.07	7.8	**1.74 ^a^**	**1.10–2.82**	9.0	1.04	0.89–1.23

^a^*p* < 0.05; ^b^ *p* < 0.01; ^c^ *p* < 0.001; N = Number of observations within category. † Percentage of cases to non-cases within each category, OR = Adjusted odd ratio, CI = 95% confidence interval. Ref. = reference category. ¥ Variable has missing observations: Taking Iron supplements = 58 (0.6%), Cooking location = 16 (0.1%), Mode of Delivery = 41 (0.4%). Not associated in the multivariate model (*p* > 0.05) = Birth order, household smoking, cooking location.
